# TreeGraph 2: Combining and visualizing evidence from different phylogenetic analyses

**DOI:** 10.1186/1471-2105-11-7

**Published:** 2010-01-05

**Authors:** Ben C Stöver, Kai F Müller

**Affiliations:** 1 Institute for Evolution and Biodiversity, University of Münster, Hüfferstraße 1, 48149 Münster, Germany; 2 Nees Institute, University of Bonn, Meckenheimer Allee 170, 53115 Bonn, Germany

## Abstract

**Background:**

Today it is common to apply multiple potentially conflicting data sources to a given phylogenetic problem. At the same time, several different inference techniques are routinely employed instead of relying on just one. In view of both trends it is becoming increasingly important to be able to efficiently compare different sets of statistical values supporting (or conflicting with) the nodes of a given tree topology, and merging this into a meaningful representation. A tree editor supporting this should also allow for flexible editing operations and be able to produce ready-to-publish figures.

**Results:**

We developed TreeGraph 2, a GUI-based graphical editor for phylogenetic trees (available from http://treegraph.bioinfweb.info). It allows automatically combining information from different phylogenetic analyses of a given dataset (or from different subsets of the dataset), and helps to identify and graphically present incongruences. The program features versatile editing and formatting options, such as automatically setting line widths or colors according to the value of any of the unlimited number of variables that can be assigned to each node or branch. These node/branch data can be imported from spread sheets or other trees, be calculated from each other by specified mathematical expressions, filtered, copied from and to other internal variables, be kept invisible or set visible and then be freely formatted (individually or across the whole tree). Beyond typical editing operations such as tree rerooting and ladderizing or moving and collapsing of nodes, whole clades can be copied from other files and be inserted (along with all node/branch data and legends), but can also be manually added and, thus, whole trees can quickly be manually constructed de novo. TreeGraph 2 outputs various graphic formats such as SVG, PDF, or PNG, useful for tree figures in both publications and presentations.

**Conclusion:**

TreeGraph 2 is a user-friendly, fully documented application to produce ready-to-publish trees. It can display any number of annotations in several ways, and permits easily importing and combining them. Additionally, a great number of editing- and formatting-operations is available.

## Background

It has become standard to apply multiple inference techniques to a given phylogenetic problem. The recent invasion of phylogenetics by Bayesian techniques (e.g., [1]), the ever improving models and algorithms for tree searches under maximum likelihood (e.g., [2,3]), and the continuously growing processor speed helped these previously computationally very expensive approaches to become a typical component of most phylogenetic studies, accompanying the widespread parsimony and distance-based approaches. At the same time, no single inference technique has consistently proven to be the single best choice. Accordingly, the researcher is well-advised to explore potential method-specific differential results, leaving him or her with the difficulty of visualizing these differences for him- or herself and for the reader. Frequently, differences are restricted to the magnitude of various measures of statistical support (such as jackknife and bootstrap proportions, Bayesian posterior probabilities), rather than being apparent from the topology. In addition, the frequently reported results from topological tests (e.g., [4]) or tracing of ancestral character states (e.g., [5]) add further importance to being able to assign a variety of numbers and graphical labels to tree nodes.

To address those needs, the first version of TreeGraph [6] had been developed, which strongly simplifies the creation of the final tree figure by the automatic positioning and formatting of multiple labels per branch. However, while one support type could directly be imported from the phylogeny inference program output, the Newick- and Nexus [7] format used by these programs precluded the direct import of more branch labels. For all additional labels (support values), the laborious work of mapping them onto the appropriate nodes remained. The cumbersome drawing part of the publication process was minimized, but it remained the user's responsibility to collect and position all information that was to be displayed at the nodes.

We figured that automating this process would be very useful, particularly so in studies of extensive gene family datasets that may contain several hundred terminals. Gene family studies using phylogenetic approaches have become a major focus with the increasing amount of available fully sequenced genomes. Typically, gene family trees suffer from weak support [8-10]. The entailed caution required when interpreting gene family trees increases the need for testing alternative inference methods, alignment methods, data partitions, and varying treatment of questionable alignment regions.

Similarly, the differential contribution of and potential conflict among different data partitions is frequently estimated by the differential success of resolution and degree of statistical support in various parts of the tree contributed by each partition [11]. This has become particularly important since multigene analysis are the rule rather than the exception, a trend further fueled by the growing availability of complete (organellar) genomes that provide easy access to a large number of genes that can be concatenated in large data matrices and then subjected to phylogenetic analyses, e.g. [12].

These trends call for a tree editor that is able to compare and ultimately visualize congruent and conflicting evidence from different analyses, while guaranteeing flexible editing and production of high-quality tree figures for publications.

## Implementation

TreeGraph 2 is written in Java and uses Swing for its graphical user interface (GUI) as well as the Apache Batik SVG Toolkit (http://xmlgraphics.apache.org/batik/), FreeHEP (http://java.freehep.org/), Java Math Expression Parser (http://sourceforge.net/projects/jep/) and BrowserLauncher (http://browserlaunch2.sourceforge.net/) libraries. Besides its GUI, which makes editing and formatting very intuitive, the current version 2 adds many features previously unavailable in the command line precursor and introduces an XML-based native file format (XTG).

## Results and Discussion

### Importing data

TreeGraph 2 can read trees in Newick or Nexus format (including additional annotations in comments specified by BEAST [13]) as well as phyloXML tree descriptions [14] and can furthermore import annotations from text files generated e.g. with a spreadsheet application. Besides that, TreeGraph 2 facilitates combining information from different phylogenetic analyses of a given dataset. This is particularly useful e.g. in the study of extensive gene family datasets with large sets of terminals. The following sections describe this feature in greater detail.

#### Mapping statistical support onto congruent nodes

For each branch of a tree opened in TreeGraph 2, the corresponding support from other trees can be mapped whenever the topology defined by the current branch is present in them. Each of these other trees may represent the result from a different analytical approach or different data partition, and support values from these trees are assigned their own label ID by which they are grouped and amenable to future formatting or editing operations. Thus, all support values that stem from a particular analysis can be individually formatted e.g. by their relative position on the branch and/or their font and style.

#### Finding conflicting nodes and mapping contradictory support

In some studies not only the support from different analyses has been mapped onto the branches but also the strongest support for a contradictory topology was determined by inspection via eye [15,16].

TreeGraph 2 uses the following algorithm automate this (for a better understanding it should be kept in mind that each branch splits a tree into exactly two subtrees).

**Figure 1:**
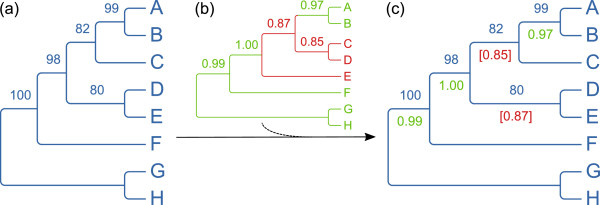
**Merging support values from different analyses - a simple contrived case**. The tree on the left (a) was first opened in TreeGraph 2 and defines the topology and optionally a first set of support values. (Alternatively a consensus tree of all analyses or any user-defined tree could be used here.) Afterwards the annotations from another tree (b) have been added which resulted in a new group of values (c) supporting (green) or contradicting (red) the initially loaded topology (blue).

Let *tree1 *specify the topology onto which contradictory support from other trees should be mapped (example in Figure 1a). For a given branch *branch1 *in *tree1*, the maximum support for a conflicting branch *branch2 *from another tree *tree2 *(example in Figure 1b) can be found as follows.

1. Find the *branch2 *which defines a subtree *subtree2 *with the smallest number of terminals that contains all leafs of a subtree *subtree1 *defined by *branch1*.

2. Inside *subtree2 *find all branches that define a subtree which are on the one hand fully enclosed by *subtree2 *and on the other hand contain at least one terminal which is also part of *subtree1 *as well as at least one leaf which is not.

3. The highest support value in the set of these branches is added as a conflicting value onto *branch1*.

This highest conflicting support value can be distinguished from congruent values by user-specified formats, e.g. brackets, asterisks or different colors (see example in Figure 1).

### Editing and formatting capabilities

The program features versatile editing and formatting options, such as automatically setting branch widths or colors according to the value of any of the unlimited number of variables that can be assigned to each node or branch.

#### Editing of node/branch data

**Figure 2:**
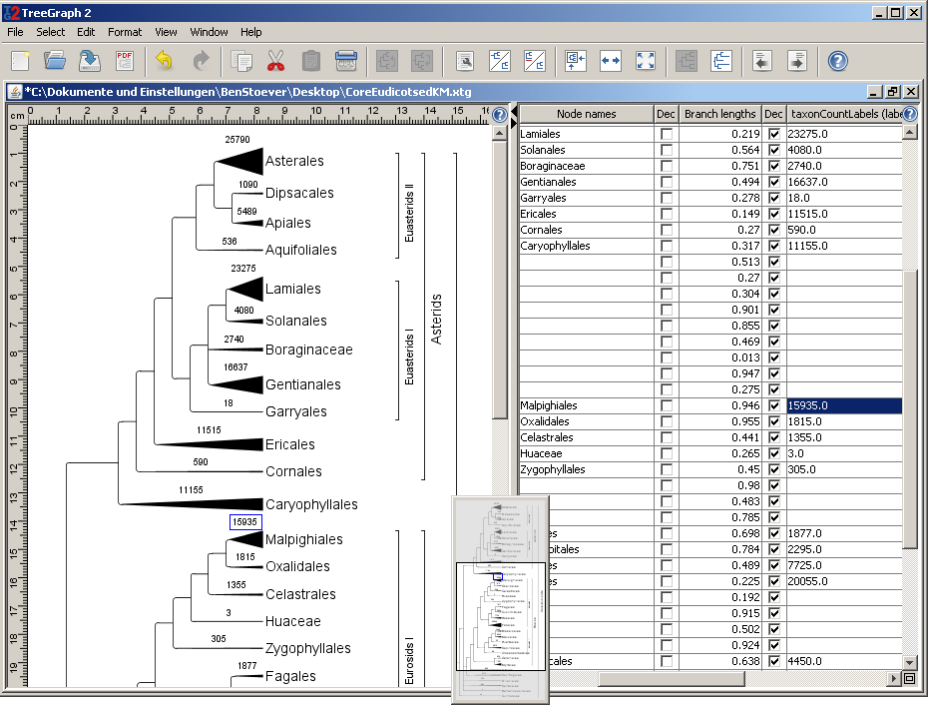
**Example view of the TreeGraph 2 GUI showing taxon counts displayed as branch widths**. The taxon counts of all terminal nodes have been imported from a table (text file) to a hidden node data column. The imported annotations have then been used as source data to set the terminal branch widths. For each TreeGraph 2 document, one can optionally view the node/branch data table in the right part of the document window as shown here.

Node/branch data imported from spread sheets or other trees (as described above), can be copied from and to other internal variables, be kept invisible or set visible and then be freely formatted (individually or across the whole tree), filtered according to their values or calculated from each other using an integrated mathematical expression parser which can access all node/branch data columns. Figure 2 shows a screenshot displaying a tree and its corresponding data table.

#### Editing operations

Beyond typical editing operations such as tree rerooting and ladderizing or moving and collapsing of nodes, whole clades can be copied or cut out and placed into new empty files or inserted (along with all node/branch data) into other trees. Since nodes can also be manually added, whole trees can quickly be manually constructed starting from an empty file.

The editing operations are facilitated by versatile additive selection options that allow selecting many elements in a tree for subsequent formatting with just a few clicks. Additionally, every operation applied to an opened tree can be easily undone or redone using the undo-function.

#### Searching, replacing and translating tree leaf names

Searching and replacing is possible across all node/branch data columns (including taxon names and node labels).

More restrictive alignment file formats do not allow lengthy taxon names, so names get truncated. In other cases, the often clumsy taxon- or lab IDs used during a study survive up to the final alignment, phylogenetic dataset and the trees constructed from it until they need to be adjusted for the final tree to be presented in a paper. TreeGraph 2 can be requested to apply a translation table to use "cleaned" taxon names for the final output. This translation table can be constructed easily with help of the data export feature and any text editor or spread sheet program. Furthermore the lab IDs (old terminal names) can be saved in a hidden data field to be able to identify the terminals by these lab IDs so that additional support values could still be added later on.

#### Formatting document elements

**Figure 3:**
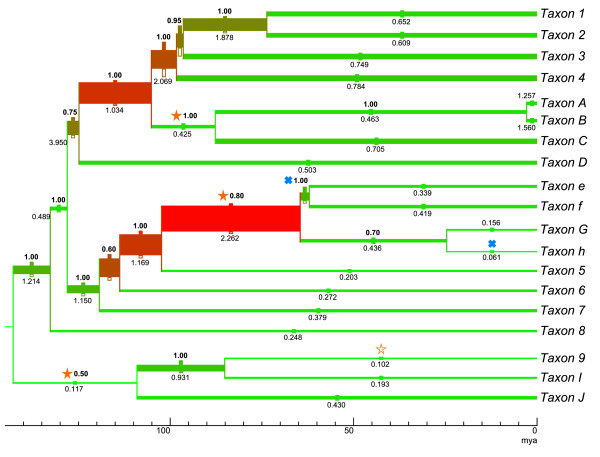
**Displaying multiple annotations and assigning element formats automatically**. The tree in this contrived example contains several annotations including ancestral divergence times (node heights; expressed as branch lengths in an ultrametric tree), DNA substitution rates, posterior clade probabilities as they could have been imported by TreeGraph 2 from, e.g., a tree file generated with help of TreeAnnotator after a BEAST analysis. As in a typical chronogram view, the age of the nodes (in million years ago) is expressed by the scale bar at the bottom. In addition, TreeGraph 2 was asked to automatically assign branch widths and line colors to illustrate the mean evolutionary rates for each branch, while the accuracy of each rate estimate was illustrated by a filled rectangular label icon above and an unfilled one below each branch (the branch width extended by the size of the upper icon describes the highest rate in e.g. a 95% confidence interval and the branch width reduced by the size of the lower icon describes the lowest rate in the interval). Text labels have been used to show the posterior clade probabilities (above the branches, bold) and the absolute substitution rates in substitutions per site per billion years (below the branches). Furthermore, this example tree contains star and cross icon labels that could be used, e.g., to highlight specific character state transitions (such as orange stars indicating "number to character" shifts (filled) or *vice versa *(not filled), and blue crosses representing "upper case to lower case" shifts).

Great flexibility is offered by the application as it allows free formatting of line- and text-formats of all document elements like nodes, branches or legends (which mark a group of terminals). Additionally branches can carry an unlimited number of textual annotations (text labels) or icons (icon labels) the color, text style or size of which can also be freely formatted (see Figure 3). All distance values in TreeGraph 2 (e.g. line width or text height) are specified in millimeters or DTP-points (1/72 inch). This feature, along with the image export function (see below), allows the user to design trees in exactly the size they should appear in print or in the exported graphic file. In addition, TreeGraph 2 offers a feature to proportionally rescale all elements of a subtree or the whole document.

#### Automatically setting line width, text height, and color

TreeGraph 2 allows automatically setting all formats (e.g. branch widths, branch colors, text colors, text heights, icon sizes) according to the value of a chosen node/branch data column. This provides a very intuitive way to graphically present the relative magnitude of, e.g., certain types of support or rates assigned to branches (see Figure 2 and 3 for examples).

### Different view modes

All editing operations are facilitated by a very convenient way to zoom in and out, fitting the zoom to the window size, and a miniature overview (Figure 2) for navigating large trees.

When applicable (i.e., given that branch length information is provided), trees can be displayed as phylogram or chronogram (Figure 3), with multiple options for adjusting a scale bar (to indicate e.g. time spans in chronograms, rates in ratograms, or branch lengths in phylograms).

### Exporting to graphic formats and printing

TreeGraph 2 outputs various vector and (anti-aliased) pixel graphic formats. Among these are SVG, PDF, or PNG, supporting transparent background where this applies. Using the graphic export function of TreeGraph 2, the most adequate graphic formats, resolutions, and image sizes for manuscripts, presentation slides, or web pages, respectively, can be specified.

### Help

An extensive, continuously updated online help system is available under http://treegraph.bioinfweb.info/Help and can also be accessed (in a context-dependent manner) from within the program. Additionally, several video tutorials are offered there to get started with TreeGraph 2 (see http://treegraph.bioinfweb.info/Help/wiki/Tutorial:Main_page).

### Comparison to previous software

**Figure 4:**
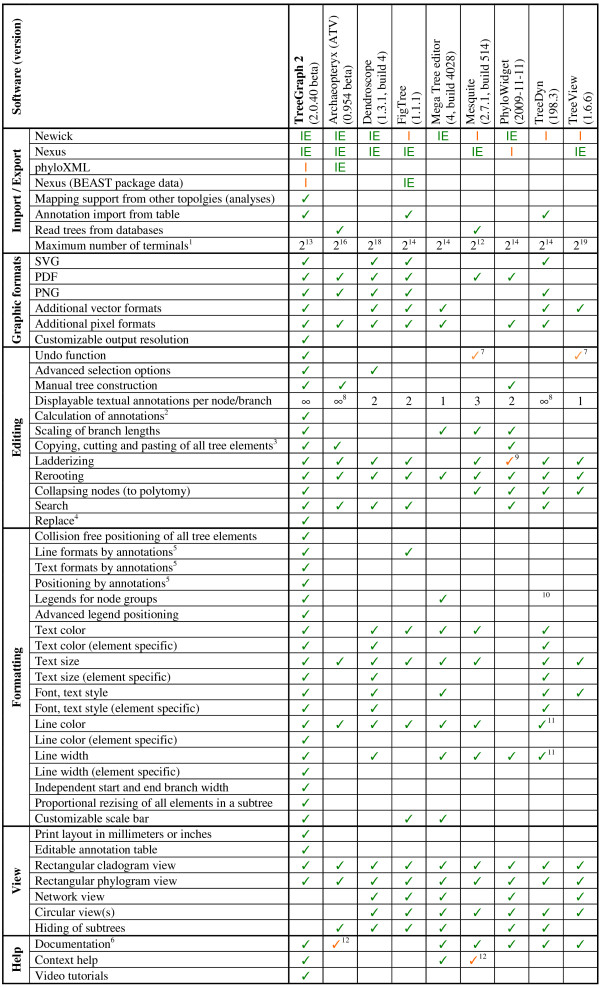
**Comparison to other tree editors**. I: Import, IE: Im- and export. ^1^All programs tested with balanced binary trees in Newick format. The value listed is the number of terminals of the largest tree that could still be opened in less than two minutes on an average desktop computer (2.2 GHz AMD Athlon™ XP processor, 1 GB RAM). ^2^Numerical and textual annotations of nodes and branches can be calculated by any user defined mathematical expression from the values of other annotations in the tree. ^3^Any tree element can be copied to any position in the same or another tree (Programs that can only copy whole trees or paste subtrees to a new file are not checked in this column.). ^4^User defined text replacement in node names and all annotations. ^5^Numerical values of annotations define formatting of tree elements (e.g. color, width, text height). ^6^Documentation going beyond the original publication and explaining the different options. ^7^Only the last edit can be undone. (In contrast, TreeGraph 2 stores a whole undo history which can be undone (and redone) to any point.). ^8^Positioning options for the labels are not offered. ^9^Only one direction (not up and down). ^10^TreeDyn allows labeling a group of nodes with a legend (not automatically positioned), but the label gets lost during edit operations like ladderizing. ^11^Specific formats for subtrees are possible. Branches and nodes cannot be formatted independently. ^12^Only very brief descriptions.

To date, a variety of tree visualization tools have been released, among which ATV [17], Dendroscope [18], FigTree (the tree editor accompanying BEAST), the MEGA tree explorer [19], Mesquite [20], PhyloWidget [21], TreeDyn [22] and TreeView [23] may be the most widely distributed. In spite of their great usefulness for the purposes they have been developed for, none of these software packages allows simultaneously visualizing, freely editing, properly formatting and exporting or printing trees with heavily annotated nodes (see Figure 4). Although TreeDyn is able to display multiple annotations on one node it is not able to automatically position them in a ready-to-publish way or to combine them from different analyses. FigTree is able to read the special Newick annotations generated by BEAST and therefore can also store several sets of annotations but only offers a limited number of ways to display them (like branch lengths or one textual annotation per branch). In contrast TreeGraph 2 (which is also able to read BEAST annotations) can show a nearly unlimited number of textual annotations at a time as well as display data in form of branch widths, line colors or many other formats.

Besides importing additional annotations from tables (which TreeDyn also offers), TreeGraph 2 is the only editor which can combine annotations (e.g. statistical support from different analysis methods) from different trees (with the same set of terminals). The information gained this way has a topological component and can therefore not simply be obtained from data in a table.

A feature closely related to the ones mentioned above is the ability to calculate numeric or textual annotations by mathematical expressions which can reference other annotations (see above). To date, a similar functionality is not offered by any other tree editor.

TreeGraph 2 features a multitude of format options which can be combined to every tree element (e.g. branches, nodes or labels) independently. As Figure 4 shows, no other tree editor currently provides functionalities like element-specific formats for all types of tree elements in combination with advanced selection options or collision free positioning of the whole tree. Moreover, none of the editors that offer at least some of TreeGraph 2's formatting options allow the user to precisely determine the print layout. In contrast to most other editors, our program offers context help buttons (which link to the online help system) everywhere in the program, making it very easy for new users to get started.

It should be noted, however, that TreeGraph 2 has been optimized as a tree editor for producing high quality tree figures and not as a viewer for trees with many thousands of taxa which could never be depicted completely in a publication or presentation. The latter is a specialty of software specifically designed for this purpose such as, e.g., Dendroscope [14] (Figure 4).

Since TreeGraph 2 is written in Java and is able to read and write all its supported formats directly from and to streams in would be possible to use it in a web application either on the server (e.g. with Apache Tomcat) or the client site (e.g. as an Java applet or a Java webstart application) to display and manipulate trees. As yet, our application would have to be integrated into such a web application by its programmer manually and we do not yet offer a ready-to-use plug-in solution for this. We do, however, offer a full documentation of our source code (including its interfaces) to facilitate such a web integration.

## Conclusions

With its easy-to-use graphical user interface and a number of semi-automatic editing and formatting options, TreeGraph 2 is a graphical editor useful in the context of any phylogenetic study. It is particularly useful where multiple, potentially conflicting trees are being produced, because its automatic combination of information from different analyses helps to identify and graphically present such incongruences. The way in which data can be imported and then assigned to nodes, manipulated or even converted to color tones, line diameters or other formats allows for a great flexibility in visualizing any kind of data associated with different parts of the tree. Together with the possibility to manually construct new clades or delete clades and the various graphic output formats supported, TreeGraph 2 greatly reduces the effort during the preparation of tree figures for presentations or publications.

## Availability and requirements

**Project name: **TreeGraph 2

**Project home page**: http://treegraph.bioinfweb.info/ (including an extensive documentation and a development section with Javadocs)

**Operating system(s): **Platform independent (Java 6 has to be available)

**Programming language**: Java

**Other requirements**: Java Runtime Environment 6.0 (or higher)

**License**: GNU General Public License

**Restrictions to use by non-academics: **none

## Authors' contributions

BCS developed TreeGraph 2, wrote the online help and contributed to the concept of the software and the manuscript. KFM was responsible for the conception and design of the software, contributed to its help system, and wrote the manuscript. Both authors have given final approval of the version to be published.
